# GSA: Genome Sequence Archive^*^

**DOI:** 10.1016/j.gpb.2017.01.001

**Published:** 2017-02-02

**Authors:** Yanqing Wang, Fuhai Song, Junwei Zhu, Sisi Zhang, Yadong Yang, Tingting Chen, Bixia Tang, Lili Dong, Nan Ding, Qian Zhang, Zhouxian Bai, Xunong Dong, Huanxin Chen, Mingyuan Sun, Shuang Zhai, Yubin Sun, Lei Yu, Li Lan, Jingfa Xiao, Xiangdong Fang, Hongxing Lei, Zhang Zhang, Wenming Zhao

**Affiliations:** 1 BIG Data Center, Beijing Institute of Genomics, Chinese Academy of Sciences, Beijing 100101, China; 2 CAS Key Laboratory of Genome Sciences and Information, Beijing Institute of Genomics, Chinese Academy of Sciences, Beijing 100101, China; 3 University of Chinese Academy of Sciences, Beijing 100049, China; 4 Collaborative Innovation Center of Genetics and Development, Fudan University, Shanghai 200438, China; 5 Center of Alzheimer’s Disease, Beijing Institute for Brain Disorders, Beijing 100053, China

**Keywords:** Genome Sequence Archive, GSA, Big data, Raw sequence data, INSDC

## Abstract

With the rapid development of sequencing technologies towards higher throughput and lower cost, sequence data are generated at an unprecedentedly explosive rate. To provide an efficient and easy-to-use platform for managing huge sequence data, here we present **Genome Sequence Archive** (**GSA**; http://bigd.big.ac.cn/gsa or http://gsa.big.ac.cn), a data repository for archiving **raw sequence data**. In compliance with data standards and structures of the International Nucleotide Sequence Database Collaboration (**INSDC**), GSA adopts four data objects (BioProject, BioSample, Experiment, and Run) for data organization, accepts raw sequence reads produced by a variety of sequencing platforms, stores both sequence reads and metadata submitted from all over the world, and makes all these data publicly available to worldwide scientific communities. In the era of **big data**, GSA is not only an important complement to existing INSDC members by alleviating the increasing burdens of handling sequence data deluge, but also takes the significant responsibility for global big data archive and provides free unrestricted access to all publicly available data in support of research activities throughout the world.

## Introduction

Next-generation sequencing (NGS) technologies have been extensively and routinely applied to a wide range of important issues in life and health sciences, leading to an unprecedented explosion in sequence data. Considering the increasingly higher throughput and lower costs attributable to rapid advancements of NGS technologies, large-scale sequencing projects for population genomics and precision medicine are ongoing or in the planning stages around the world, *e.g.*, the US Precision Medicine Initiative (PMI) [1], UK10 K Project [2], Icelandic Population Genome Project [3], and Dog 10 K Project [4]. As a corollary, such deluge of sequencing data poses great challenges in big data deposition, integration, and translation [5], [6]. Accordingly, it is fundamentally crucial to store and manage sequencing data in support of integrative in-depth analyses and large-scale data mining.

The International Nucleotide Sequence Database Collaboration (INSDC) [7] operating between the DNA Data Bank of Japan (DDBJ) [8], the European Molecular Biology Laboratory-European Bioinformatics Institute (EMBL-EBI) [9], and the National Center for Biotechnology Information (NCBI) [10], provides valuable services for archiving a broad spectrum of sequence data. However, with the exponentially accumulating volume of sequence data, submitting big data to INSDC database resources becomes increasingly daunting and time-consuming, simply because network bandwidth is a formidable bottleneck for big data transfer across countries/regions. This situation is particularly severer in China; to our experience, for instance, submission of ∼1 terabyte (TB) data to the NCBI Sequence Read Archive (SRA) takes ∼2 weeks based on the 150-Mbps upload bandwidth over a shared international network in Beijing Institute of Genomics (BIG), Chinese Academy of Sciences (CAS). China, with the increasing funding support in biomedical research, has been a powerhouse in generating enormous amounts of sequencing data. Given the huge population and rich biodiversities in China, it is undoubted that data generated from sequencing projects for the Chinese population (*e.g.*, CAS PMI at http://news.xinhuanet.com/english/2016–01/09/c_134993997.htm) and domestically featured species would be growing strikingly at extraordinarily exponential rates, which accordingly brings an insurmountable challenge and burden to current practice of data submission and sharing.

To address this issue, here we present Genome Sequence Archive (GSA; http://bigd.big.ac.cn/gsa or http://gsa.big.ac.cn), a data repository for archiving raw sequence data. As a core database resource of BIG Data Center [11] (http://bigd.big.ac.cn), GSA is built based on INSDC data standards and structures and provides data archival services for scientific communities not only in China but also throughout the world. GSA accepts raw sequence reads produced by a variety of sequencing platforms, stores both sequence reads and metadata, and provides free and unrestricted access to all publicly available data for worldwide scientific communities.

## Implementation

GSA is implemented with Java Server Pages (JSP; a Java programming framework for constructing dynamic web pages), Spring (an application framework and inversion of control container; http://www.springsource.org), Struts (a Model-View-Controller framework for creating Java web applications; http://struts.apache.org), and MyBatis (a persistence framework for the database connection and operation; http://www.mybatis.org). GSA adopts MySQL (http://www.mysql.org) as relational database management system to store metadata information. All codes are developed using Eclipse (http://www.eclipse.org), an integrated development environment (IDE) that features rapid development of Java-based web applications. To provide stable web services, GSA is hosted on a CentOS-7 operating system with four servers, namely, Apache serving static content, Tomcat serving dynamic content, a MySQL server for database management, as well as a FTP server for file upload and download.

## Database content and usage

### Data structure and organization

**Figure 1:**
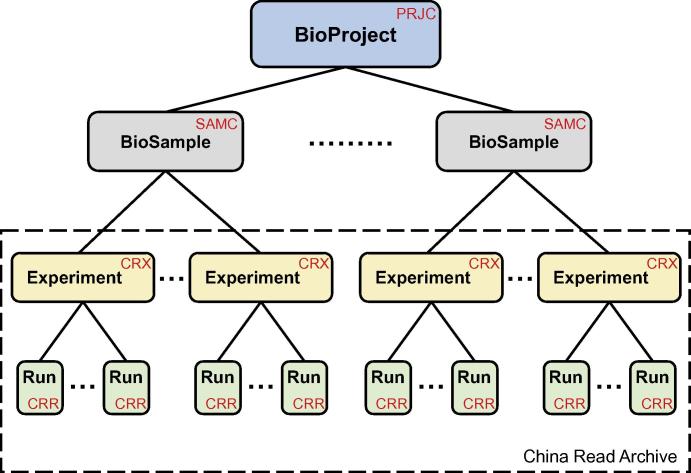
Data model in GSAPrefixes of accession numbers for data objects, including BioProject, BioSample, Experiment, and Run, are indicated in red. Data objects Experiment and Run constitute China Read Archive.

Designed for compatibility, GSA follows INSDC data standards and structures. All data are organized into four objects, *i.e.*, BioProject, BioSample, Experiment, and Run (Figure 1). “BioProject”, bearing an accession number prefixed with “PRJC” (where C, hereinafter, stands for China), provides an overall description for an individual research initiative, including basic description, organism, data type, submitter, funding information, and publication(s) if available. “BioSample”, possessing an accession number prefixed with SAMC, contains descriptive information about biological materials used in the experiments, including sample types and attributes. “Experiment”, having an accession number prefixed with CRX, provides a detailed description of treatments for a specific BioSample, including experiment intention, library method, and sequencing type. “Run”, adopting an accession number prefixed with CRR, includes a list of sequence data file(s) related to a specific experiment. It is noted that “Experiment” and “Run” constitute China Read Archive (CRA). Based on these standardized data objects, GSA not only facilitates data submission and deposition, but also enables data sharing and exchange.

In addition, GSA features umbrella projects and provides an organizational structure for a large collaborative project consisting of multiple sub-projects that are funded by a same grant and have very close collaborations. GSA is well supported by CAS that functions as the national scientific think tank and academic governing body. Currently, two umbrella projects from CAS Strategic Priority Research Programs and one CAS Key Research Program make it officially mandatory to submit sequencing data to GSA.

### Data archive and statistics

**Figure 2:**
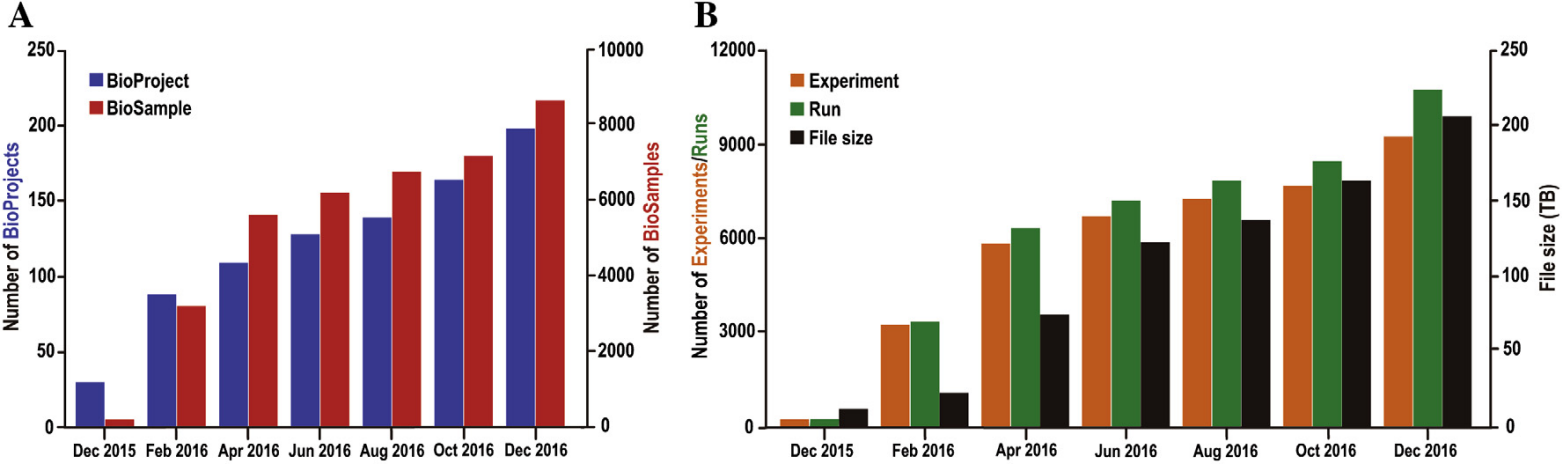
Data statistics of GSA**A.** Numbers of BioProjects and BioSamples in GSA. **B.** Numbers of Experiments and Runs, as well as file size in GSA. All statistics are based on data submissions ranging from December 2015 to December 2016.

GSA accepts data submissions from all over the world, covers the spectrum of sequence reads generated by a variety of sequencing platforms, and accommodates several commonly-used file formats, like FASTQ, BAM, and VCF. GSA performs validations for all submitted data items to ensure data integrity and increase data reusability. Similar to INSDC members, GSA allows users to set data as either public or controlled, indicating that the data is publicly accessible or placed under controlled access over a given period of time, respectively. Regarding data security, all submitted data have copies stored in physically separate disks. Since its inception in August 2015, GSA presents a dramatic increase on data submissions in terms of the numbers of BioProjects and BioSamples, Experiments, and Runs, as well as file size (Figure 2). As of December 2016, GSA houses a total of 198 BioProjects, 8674 BioSamples, 9263 Experiments and 10,745 Runs for more than 80 species, submitted by more than 160 data providers from a total of 39 institutions, and archives more than 200 TBs of sequence data.

### Data submission and retrieval

**Figure 3:**
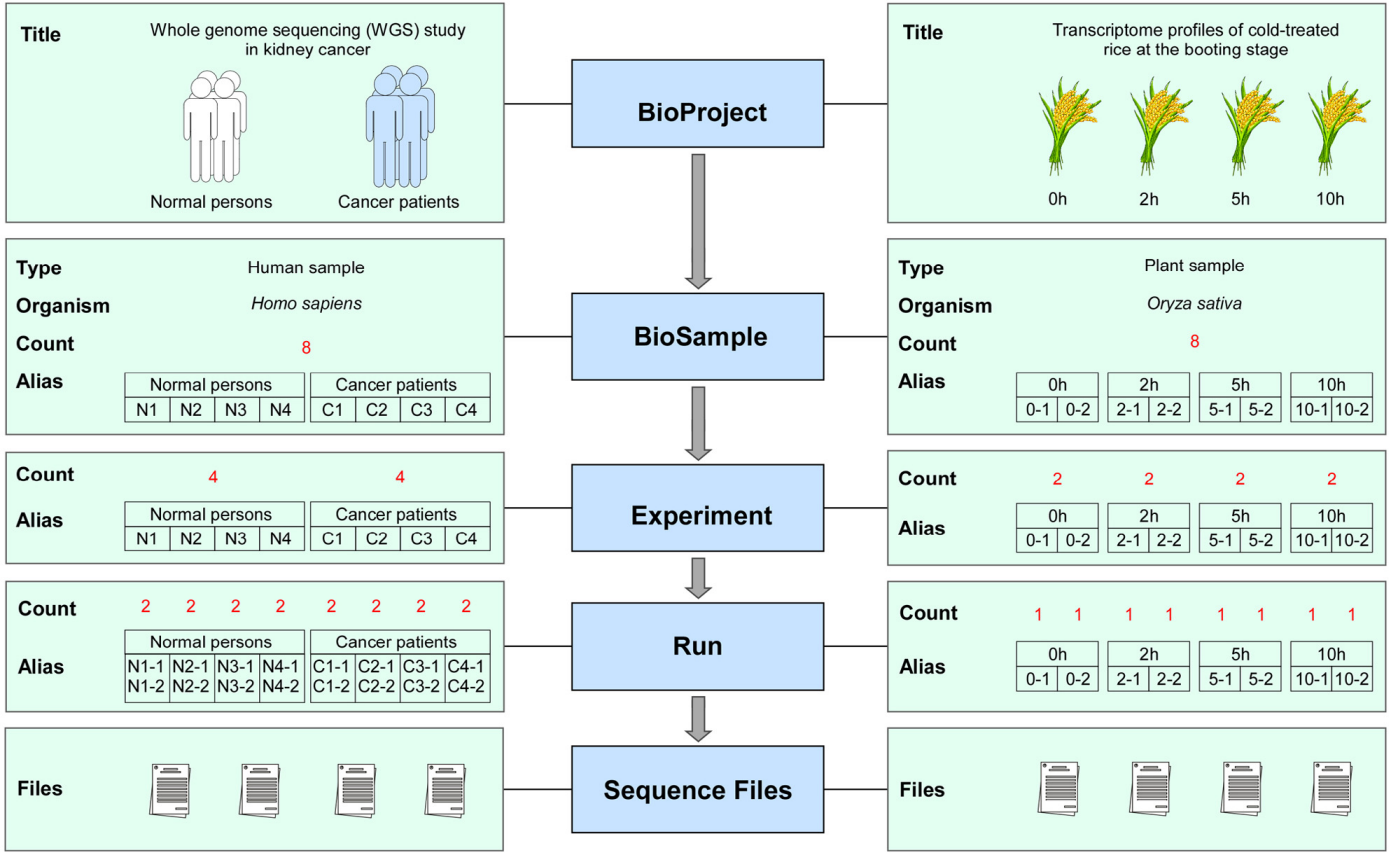
Graphic illustration of data submissions to GSATwo representative studies are provided here as examples to depict the data objects involved in data submission.

To create a submission, users need to register and log into the GSA system. Basically, to submit data to GSA, there are five straightforward steps involving BioProject, BioSample, Experiment, Run, and Sequence Files (Figure 3). In order to maximally simplify the submission procedure, GSA is equipped with a user-friendly input wizard for metadata collection. To ease sequence file uploading, GSA provides a FTP server supporting two Internet Protocols (IPv4 and IPv6). In addition, GSA provides user-friendly web interfaces for data query and browsing. Users can search the data of interest by specifying a given BioProject, BioSample, Experiment, or Run ID. Moreover, GSA allows users to conduct advanced search by inputting species name, sequencing type, sequencing platform, disease/phenotype/trait, tissue/cell line, *etc*. GSA also allows users to browse all publicly available BioProjects, BioSamples, and Experiments.

## Perspectives and concluding remarks

“*With great power comes with great responsibility*”. Nowadays, China is the second largest economy, playing an increasingly important and influential role in the global economy. Equally, in academia, it is time for us to implement the practice of archiving sequence data for worldwide scientific communities, especially considering the larger quantities of sequence data generated in China. Equivalent to INSDC members, GSA is committed to archiving raw sequence data. GSA’s ultimate goal, which is also the expectation from funding agencies, is to provide free archival services for raw sequence data, establish and promote a centralized archival practice in China, play an important role in global sequence data archive, and support research activities in both academia and industry throughout the world. In addition, there are also strong domestic incentives and agreements from academia, industry, and government (over 1000 supporters from more than 380 organizations; http://bigd.big.ac.cn/gdsd) to deposit data into GSA and make GSA a centralized archival resource in China.

To sum up, GSA is a data repository for archiving raw sequence data. Designed for compatibility, GSA adopts INSDC data standards and structures, archives both sequence reads and metadata submitted from all over the world, and makes all these data publicly available to worldwide scientific communities. In the era of big data, GSA is not only an important complement to existing INSDC members by alleviating the increasing burdens of handling sequence data deluge, but also takes the significant responsibility for global big data archive and provides free unrestricted access to all publicly available data in support of research activities throughout the world. In future, we will not only upgrade infrastructure of GSA to achieve big data storage, exchange and sharing, but also will develop new functionalities to archive population-based PMI data and a variety of metagenome data.

## Authors’ contributions

WZ, ZZ, HL, and XF conceived of the idea and supervised the project. WZ, YW, and BT designed the system architecture. YW, JZ, FS, YY and ZB wrote the source code. QZ, ND, TC and XD tested the system. TC, LD and SSZ conducted data quality control and provided feedback service. HC, MS, YS, SZ, LL and LY constructed and maintained the network and hardware infrastructure. WZ, SSZ, and ZZ drafted the manuscript. ZZ, WZ and JX revised the manuscript. All authors read and approved the final manuscript.

## Competing interests

The authors have declared no competing interests.
